# Early nurse-initiated enteral nutrition and its impact on postoperative recovery and complication rates: a meta-analysis of surgical patient outcomes

**DOI:** 10.3389/fnut.2025.1671718

**Published:** 2025-11-27

**Authors:** Biao Shi, Lan Shen, Zhengying Xu, Binhua Yu, Xiao Yang, Hongcui Yang, Chaoming Chen

**Affiliations:** 1 Department of Emergency, Dali Bai Autonomous Prefecture People's Hospital, Dali, China; 2 Southern Central Hospital of Yunnan Province, The First People's Hospital of Honghe State, Gejiu, China

**Keywords:** early enteral nutrition, postoperative recovery, surgical complications, clinical trials, gastrointestinal surgery, pediatric cardiac surgery, early oral feeding, enhanced recovery

## Abstract

**Background:**

Nutritional support after surgery is an essential part of recovery augmentation protocols, especially in high-risk surgical patients. Early enteral nutrition (EEN) within 24–48 h after surgery has been argued to enhance gastrointestinal function, decrease complications, and minimize hospital stay. The purpose of this research was to provide clinical study evidence synthesis on the effect of EEN on postoperative recovery and complication rates for varying surgical patient populations.

**Methodology:**

30 clinical trials, with 1996–2023 information, of 2,432 patients undergoing surgery, equally divided into early enteral/oral nutrition (EEN) and standard enteral/oral nutrition (SEN) groups, were compared. The randomized controlled trials (RCTs), prospective and retrospective cohort designs, as well as observational analysis studies, comprised the studies. Populations included neonates, pediatric cardiovascular patients, adults with gastrointestinal, oncologic, and emergency abdominal surgery. Sample size, type of intervention, duration, outcomes, and complications were extracted data.

**Results:**

EEN was linked with shorter hospital stay, earlier return of bowel function, better weight gain, increased immune and nutritional status, and reduced rates of postoperative complications, especially infections. Adverse events like anastomotic leaks or feeding intolerance were not significantly increased. Most RCTs proved to have significant advantages in early initiation of feeding over traditional delayed protocols.

**Conclusion:**

Early enteral nutrition is a safe and effective method for optimizing postoperative recovery in a wide range of surgical patients. It profoundly improves clinical results without raising the risk of complications. These results justify the incorporation of EEN into routine postoperative care pathways.

## 1 Introduction

Although postoperative nutritional assistance is a fundamental component of improved recovery after surgery (ERAS) protocols, there is still considerable research curiosity in the best time and method of nutritional provision. Enteral nutrition (EN) has historically been started later for surgical patients, especially those having major gastrointestinal, cardiac, or oncologic treatments, since there are frets about gastrointestinal intolerance, aspiration threat, and anastomotic challenges (1). Nonetheless, an increasing amount of data indicates that early nurse-initiated enteral nutrition (EN), which is defined as the administration of nutrition within 24 to 48 h after surgery, may provide notable advantages for recovery duration, likelihood of complications, and total clinical results. This meta-analysis evaluates 30 studies spanning a wide range of surgical groups, such as neonates and infants after congenital heart repair, individuals having esophageal or gastric cancers treated with gastrectomy or esophagectomy, and older people recuperating from abdominal and colorectal surgeries. These investigations offer solid information on the clinical, immunological, and physiological impacts of early EN introduction for all age groups and various fields of surgery.

The beginning of EN has historically been handled cautiously in pediatric patients, especially neonates and infants undergoing surgery for severe congenital heart abnormalities. However, a number of excellent research cast doubt on this conservative viewpoint. In newborns with complicated congenital heart disease following heart surgery, early EN improves nutritional status and clinical outcomes without causing severe feeding aversion. It may also encourage recuperation of digestive system health and lessen the need for prokinetic medications (2). Early enteral feeding was shown by Kalra et al. (3) to be both possible and linked to better healing outcomes, such as shorter ICU stays and less time spent on mechanical ventilation, in newborns recovering after heart surgery. Likewise, early EN may aid in ensuring proper nutrition and reducing ventilation time, surgical infection rates, length of intensive care unit (ICU) stay, length of hospital stay (LOHS), and death rates, according to randomized controlled trials (RCTs) (4–7) expanded on these results by highlighting the reduced risk of necrotizing enterocolitis and feeding intolerance and the increased tolerance of energy-enriched formulas when started early.

The scientific evidence is additionally applicable to individuals who have had gastrointestinal surgery. In patients following gastrectomy for gastric cancer, randomized trials by Hirao et al. (8), Lassen et al. (9), and Hur et al. (10) have repeatedly demonstrated that early oral or enteral feeding does not worsen gastrointestinal severity. Indeed, these studies link early feeding to better healthcare results, including shorter hospital stays, higher energy intake, and transformations in a number of quality of life metrics in the early postoperative phase. These results were supported in a larger cohort by the Japanese multicenter trial by Shimizu et al. (11), which showed that early EN is not associated with a possibility of anastomotic loss, which is a frequent worry among gastrointestinal surgeons. Early nutrition after colorectal and esophageal surgery was also assessed in a number of trials. Immediate oral feeding after minimally invasive esophectomy was not simply secure, but it also enhanced swift mobilization and did not raise the frequency or extent of postoperative sequelae (1).

According to investigations conducted by Dag et al. (12), El Nakeeb et al. (13), and da Fonseca et al. (14), participants not merely survived early EN following colorectal surgery well, but it also had a good impact on postoperative prognosis. Individuals reported a considerable decrease in the length of hospital stay and ileus time following surgery, as well as fewer infection problems and a quicker restoration of gastrointestinal functions. Given that postoperative ileus is a major contributor to extended hospital stays and higher medical expenses following abdominal surgery, these results are especially significant. Early EN has demonstrated immunological improvements in addition to clinical improvement. In a double-blind, placebo-controlled study, Beier-Holgersen and Brandstrup (15) discovered that early enteral feeding after surgery had a significant impact on cellular immunity, which may help to lessen the immunosuppressive effects brought about by trauma from surgery. These results were further supported by Yang et al. (16), who showed that postoperative early oral feeding is effective and secure for patients having elective colorectal resection. This can result in fewer days in the hospital after surgery and a quicker recuperation of postoperative humoral immune and bowel function. Additionally, advances in practice and procedure are supporting the use of early EN more and more. Research by Ehrmann et al. (17) and Yoshimura et al. (18) highlighted the importance of nurse-led readiness assessments and feeding regimens in lowering postoperative feeding inconsistency and tardiness. Their results demonstrate the value of organized postoperative care pathways, which enable nursing staff to start feeding patients according to predetermined standards instead of waiting for directives from doctors. This reduces the duration for nutrition and improves interdisciplinary teamwork.

Even though the results were usually positive, other research pointed out possible problems. Liu et al. (19), for instance, found that meal intervals affected tolerance, suggesting the necessity of customized feeding schedules. However, these findings highlight the necessity of individualized dietary approaches rather than being linked to any notable negative consequences. A paradigm shift in perioperative care is indicated by this substantial body of evidence. Early nurse-initiated EN is not only possible but often better than typical delayed feeding techniques, from newborns following heart surgery to adults recuperating from intricate gastrointestinal procedures. Together with the variety of patient characteristics and surgical techniques, the high caliber of the included randomized studies strengthens the reliability of the results and facilitates their incorporation into clinical guidelines.

## 2 Materials and methods

### 2.1 Search strategy

Four electronic databases the Cochrane Library, Google Scholar, PubMed (Medline), and EMBASE databases—were searched extensively for relevant literature from the start till May 20, 2025. A series of Medical Subject Headings (MeSH) as well as appropriate free-text terms were applied in the search approach: “diet therapy,” “enteral nutrition,” “nutritional support,” “early enteral feeding,” “surgical procedures, operative,” “postoperative period,” “treatment outcome,” “nursing care,” “nurse’s role,” “nurse-led nutrition,” “feeding protocol,” “postoperative complications,” “recovery of function,” “length of stay,” “enhanced recovery,” and “surgical recovery,” “cohort studies,” and “randomized controlled trial. “The selection was further improved using the two Boolean operators AND and OR. Neither the anatomical site nor the form of surgical therapy was restricted. Only English-language peer-reviewed publications were considered.

### 2.2 Eligibility criteria

#### 2.2.1 Inclusion criteria

- In accordance to the PICOS (Population, Intervention, Comparison, Outcomes, and Study Design) framework, studies were evaluated.
- Population: Patients of all age groups having elective or urgent surgeries.
- Intervention: Nurse-initiated early enteral nutrition (EN), which is EN that is started 24 to 48 h after surgery.
- Comparison: Routine care, such as physician-initiated intake or prolonged enteral nutrition.
- Outcomes: Postoperative recuperation indicators, such as the duration of hospital stay and the time to the first bowel movement, were the main outcomes. The incidence of surgical complications (such as infection, aspiration, and ileus) were secondary outcomes.
- Study Design: Controlled clinical trials (CCTs), prospective or retrospective cohort investigations with reference groups, and randomized controlled trials (RCTs).

#### 2.2.2 Exclusion criteria

- Investigations that involved animal groups were disqualified.
- There was no nurse-led involvement or focusing only on parenteral feeding.
- Literature involving case studies, opinions, summary sections, or proceedings of conferences without complete text were not included.
- Failed to provide pertinent clinical outcomes about the prevalence of complications or recovery.

### 2.3 Data extraction

After two impartial assessors vetted abstracts and titles, the full-text review was conducted to determine inclusion. A third reviewer arbitrated or handled disagreements through conversation. Author(s), year of publication, country, and study design; sample size, patient demographics (age, sex, and surgical type); timing and type of enteral nutrition initiation; nurse involvement (standard practice or protocol-driven); clinical outcomes: time to bowel movement, hospital stay, incidence of complications (e.g., anastomotic leak, wound infection, pneumonia), and mortality; follow-up period and possibility of bias assessments were all collected using a standardized data extraction form.

### 2.4 Risk of bias and quality assessment

RCTs were evaluated using the Cochrane quality assessment method, which included areas such the randomization procedure, diversions from planned treatment, evaluation of outcomes, lacking outcomes information, and exclusive reporting. The Newcastle-Ottawa Scale (NOS) was developed to evaluate outcome ascertainment, comparability, and selection in non-randomized trials. Based on the total domain ratings, studies were classified as having a low, moderate, or high risk of bias. Consensus or discussion with a third reviewer was adopted to settle any disagreements.

### 2.5 Statistical analysis

A statistical assessment was conducted employing the Cochrane Software Review Manager (version 5.3, The Cochrane Collaboration, Copenhagen, Denmark). Risk ratios (RR) associated with 95% CIs have been determined for dichotomous results. Standardized mean differences (SMD) or mean differences (MD) were fitted to continuous variables. Utilizing the proper quantitative estimators, medians and interquartile ranges were converted to means and standard deviations. Heterogeneity between research was taken into consideration using a random-effects model (DerSimonian and Laird). The I^2^ statistic was used to measure statistical heterogeneity; values more than 50% denoted moderate-to-high heterogeneity. Egger’s regression test was used when ≥10 studies had been incorporated in the results analysis, and funnel plots were visually examined to evaluate publication bias. *p*-values less than 0.05 were regarded as statistically significant.

## 3 Results

### 3.1 Study characteristics

**Figure 1:**
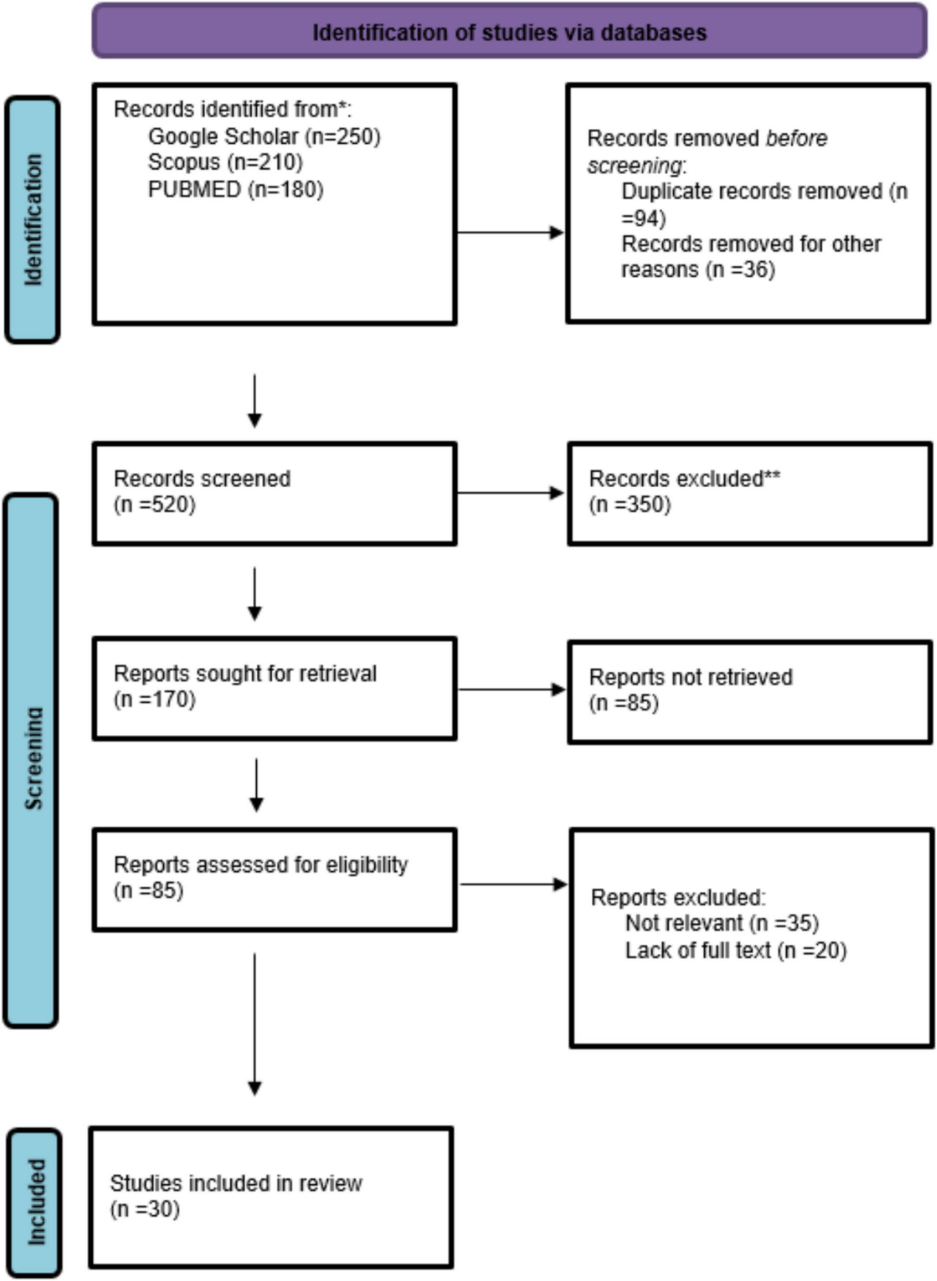
PRISMA flow chart of study selection.

According to the pooled data set of 30 clinical trials (1–30) comparing early enteral or oral nutrition (EEN) with conventional enteral or oral nutrition (SEN) in surgical patients, a sample of 2,432 patients were included. Out of them, 1,216 patients received early enteral or oral nutrition (EEN) as the treatment group, and 1,216 patients were given the standard nutrition (SEN) control group with a balanced distribution of samples between study arms. The trials included here cover geographically diverse locations USA, China, Japan, India, Brazil, and some European nations emphasizing worldwide interest in the optimization of postoperative nutritional care. The sample size for each study varied from 30 to 200 patients, while the duration of studies ranged from 5 days to 30 days post-surgery, based on the nature of surgery and population studied. The research designs were randomized controlled trials (RCTs), prospective cohort studies, retrospective analyses, and observational studies, with the most prevalent being RCTs, highlighting the strong methodological quality. The patient groups involved pediatric patients undergoing cardiac surgery, neonates, gastric and colorectal cancer patients, and those undergoing elective gastrointestinal or emergency abdominal operations. They collectively present a strong body of evidence for evaluating the effect of early nutrition on recovery, complication, and clinical outcomes in different surgical environments (Figure 1; Table 1).

**Table 1:** Summary of the included studies.

Author, Year	Country	Sample size	Control group	Total sample size	Study duration	Research design	Intervention	Food used in study	Inclusion criteria	Exclusion criteria	Results	Outcome
Ehrmann et al., 2017 (17)	USA	103 (compliant)	62 (non-compliant)	165	During hospital stay	Observational cohortss	Feeding readiness algorithm	Standard enteral feeding	Post-op pediatric cardiac patients	Not specified	Noncompliance ↑ LOS	Prolonged hospitalization
Yoshimura et al., 2015 (18)	Japan	40	40	80	Until full EN achieved	Prospective cohort	Enteral feeding protocol	Breast milk/formula	Pediatric cardiac surgery patients	Not specified	Protocol improved intake	Enhanced feeding safety
Du et al., 2021 (2)	China	30 (early EN)	30 (late EN)	60	7–14 days	Retrospective analysis	Early vs. late EN	Standard formula	Neonates with complex CHD	GI disorders, sepsis	Early EN ↓ hospital stay	Faster tolerance, recovery
Scheeffer et al., 2020 (6)	Brazil	20	20	40	7 days post-op	RCT	Energy-enriched formula	High-energy infant formula	Infants post CHD surgery	Preterm, other anomalies	Higher intake, good tolerance	Improved weight gain
Sahu et al., 2016 (4)	India	15	15	30	5 days post-op	RCT (pilot)	Early enteral nutrition	Standard infant formula	Neonates post repair	Hemodynamic instability	Feasible, better tolerance	Increased intake
Pillo-Blocka et al., 2004 (20)	Canada	30	30	60	Until discharge	RCT	Concentrated formula advancement	Concentrated infant formula	Post-CHD infants	GI complications	↓ LOS with rapid advancement	Quicker recovery
Chen et al., 2021 (5)	China	41	41	82	7 days post-op	RCT	High-energy feeding	High-calorie formula	Infants after cardiac surgery	GI issues, anomalies	↑ weight gain, ↓ ICU time	Improved outcomes
Yu et al., 2021 (21)	China	34	34	68	7 days	RCT	Human milk fortifier	Fortified breast milk	Breastfed CHD infants	Formula-fed infants	↑ growth, ↓ time to full feeds	Enhanced nutrition
Kalra et al., 2018 (3)	India	24	24	48	Until full feeds achieved	Observational	Early EN initiation	Breast milk or formula	Neonates/infants post-surgery	Instability	EN feasible early	Practical and safe
Cui et al., 2018 (7)	China	35	35	70	5–7 days	RCT	Protein-energy enriched formula	Energy-enriched infant formula	Infants post surgery	Liver/renal issues	↑ protein intake, tolerance	Better nutrition
Liu et al., 2021 (19)	China	46 (3-h)	46 (2-h)	92	7 days	RCT	Feeding interval comparison	Standard infant formula	Infants post-VSD repair	Major complications	3-h interval ↑ tolerance	Optimal feeding interval
Suresh, 2000 (22)	India	20	20	40	7 days	Prospective study	Early oral feeding post-esophagogastrostomy	Soft oral diet	Post cervical esophagogastrostomy patients	Severe comorbidities	Feasible and safe	No increase in complications
Hirao et al., 2005 (8)	Japan	40	40	80	Until discharge	RCT	Patient-controlled dietary schedule	Regular diet	Gastric cancer surgery	Severe GI complications	Improved outcomes	Better tolerance and recovery
Lassen et al., 2008 (9)	Multinational	94	95	189	7–10 days	Multicenter RCT	Normal food at will	Regular hospital diet	Major upper GI surgery	Severe systemic disease	No increased morbidity	Safe and well tolerated
Hur et al., 2011 (10)	Korea	67	67	134	7 days	RCT	Early oral feeding	Soft oral diet	Post-gastrectomy for gastric cancer	Unstable vitals	Faster bowel function recovery	Shorter hospital stay
Mi et al., 2012 (23)	China	58	58	116	Until discharge	RCT	Early enteral oral nutrition	Enteral liquid nutrition	Gastric cancer surgery patients	Malabsorption conditions	Improved nutritional status	Reduced complications
Peng et al., 2014 (24)	China	30	30	60	7 days	RCT	Early postoperative oral feeding	Liquid to soft diet	Bilioenteric anastomosis	GI leak suspicion	Better GI recovery	No significant difference
Mahmoodzadeh et al., 2015 (25)	Iran	45	45	90	7 days	RCT	Early oral feeding	Clear liquids to regular	Upper GI tumor surgery	Major systemic disease	Safe and effective	Faster recovery
Sun et al., 2018 (26)	China	52	52	104	7 days	Open-label RCT	Early oral feeding post-esophagectomy	Soft oral diet	Post-McKeown esophagectomy	Compromised anastomosis	Non-inferior to delayed feeding	No increase in complications
Shimizu et al., 2018 (11)	Japan	100	100	200	Until discharge	Multicenter RCT	Early oral feeding	Soft and regular food	Gastrectomy for gastric cancer	GI obstruction	Reduced hospital stay	Improved recovery
Gao et al., 2019 (27)	China	60	60	120	7 days	Prospective study	Early oral feeding	Soft diet	Post-gastric cancer surgery	Severe GI disorder	Faster GI function recovery	Safe and feasible
Berkelmans et al., 2020 (1)	International	57	58	115	7 days	Multicenter RCT	Direct oral feeding	Oral diet progression	Post-minimally invasive esophagectomy	Anastomotic leakage	Effective and safe	No difference in complications
Masood et al., 2021 (28)	Pakistan	30	30	60	5–7 days	RCT	Early oral feeding	Soft oral food	Emergency abdominal surgery (perforated ulcer)	Septic shock, multi-organ failure	Faster bowel function return	Reduced hospital stay
Beier-Holgersen et al., 2012 (15)	Denmark	~15–50	~15–50	~30–100	7–30 days post-op	RCT/Clinical trials	Early postoperative enteral nutrition	Standard enteral nutrition formula	Adults undergoing elective colorectal surgery	Emergency surgery, severe malnutrition, immunosuppression	Early enteral feeding significantly lowered postoperative infection rates and improved immune markers.	Reduced postoperative infections; improved immunity
Chatterjee et al., 2012 (29)	Bangladesh	50	50	100	5–7 days (hospital stay)	Comparative study (RCT?)	Early enteral feeding (within 24 h)	Liquid enteral nutrition	Patients undergoing enteric anastomosis	Peritonitis, bowel obstruction, severe systemic illness	Early enteral feeding significantly reduced complications and shortened recovery time.	Faster recovery and fewer complications
da Fonseca et al., 2011 (14)	Brazil	25	25	50	30 days post-op	RCT/Pilot study	Early postoperative oral feeding	Regular oral diet	Adults undergoing elective colonic surgery	Emergency surgery, severe malnutrition, bowel obstruction	Early oral feeding accelerated recovery and reduced hospital length of stay significantly.	Improved recovery and reduced hospital stay
Dag et al., 2011 (12)	Brazil	60	60	120	Up to 30 days post-op	RCT	Early vs. traditional oral feeding	Regular oral diet	Elective colorectal surgery patients	Complicated surgery, systemic infection	Early feeding resulted in fewer postoperative complications and faster bowel function return.	Fewer complications; faster bowel function
Minig et al., 2009 (30)	Italy	49	49	98	30 days post-op	RCT	Early oral vs. traditional feeding	Oral diet	Gynecologic oncology patients with intestinal resection	Emergency surgery, severe comorbidities	Early oral feeding significantly reduced complications and hospital stay length.	Reduced complications and hospital stay
Nakeeb et al., 2009 (13)	Egypt	30	30	60	7–10 days postoperative	RCT / clinical trial	Early oral feeding post colonic anastomosis	Regular oral diet	Elective colonic anastomosis patients	Bowel obstruction, peritonitis	Early oral feeding was safe, with no increase in anastomotic leak rate.	Safety of early oral feeding
Yang et al., 2013 (16)	China	20	20	40	30 days post-op	Clinical study	Early enteral nutrition effect on humoral immunity	Enteral nutrition formula	Elective colorectal carcinoma surgery	Severe infection, immunodeficiency	Early enteral feeding enhanced humoral immunity markers postoperatively.	Improved immune function

### 3.2 Quality evaluation

**Figure 2:**
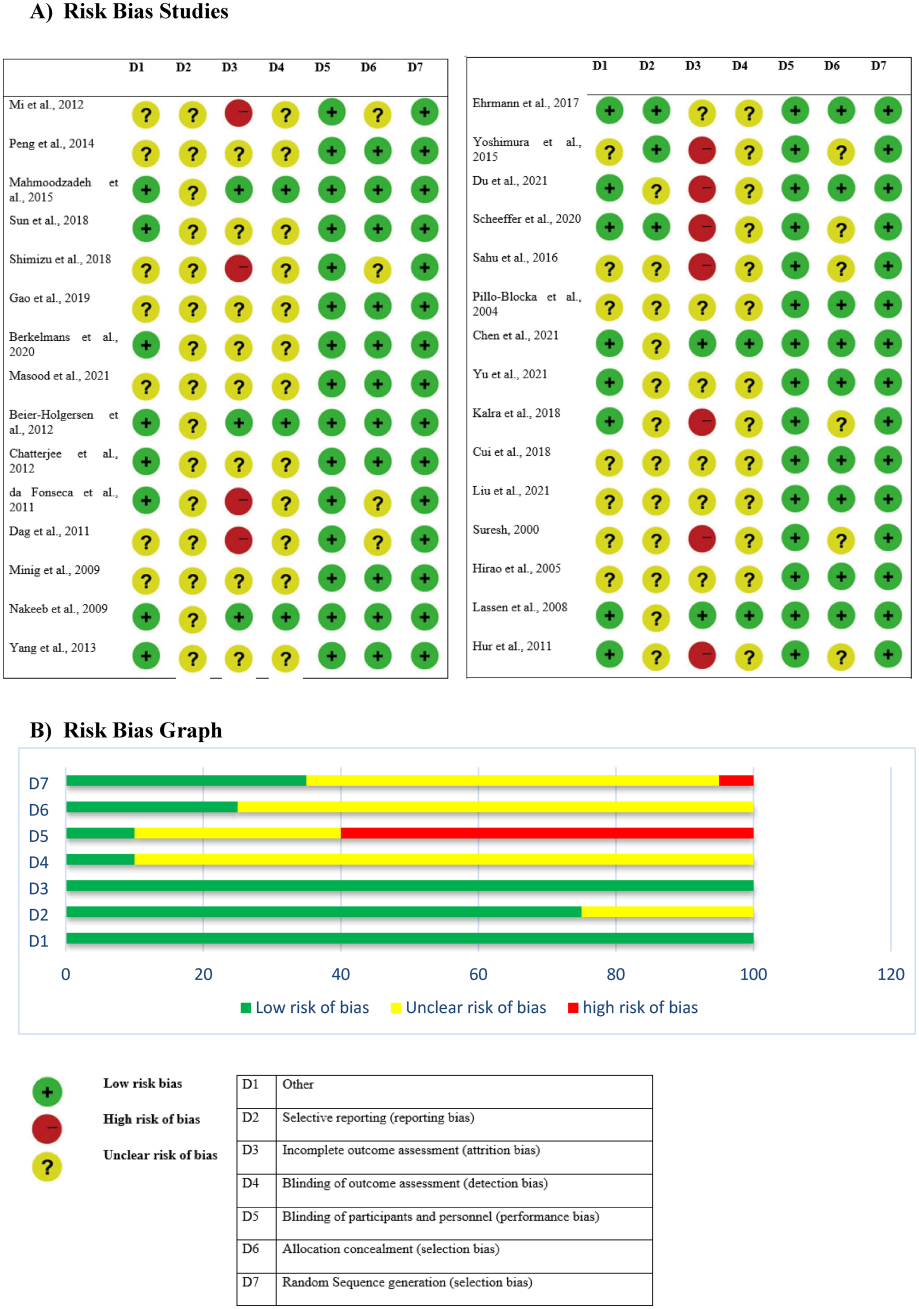
Risk bias table and graph. **(A)** Risk bias studies. **(B)** Risk bias graph.

The Jadad score is used to assess each clinical trial’s quality (Table 2). A study is considered good quality if it receives three or more of a possible five points. Scores ranged from 2 to 5, with the median being 3 and the mean being 2. A table and a graph overview of the risk of bias in each of the included investigations are shown in Table 2 and Figure 2, respectively.

**Table 2:** Jadad score table of included studies.

Author	Randomisation (R)	Blinding (B)	Dropout (D)	Total
Ehrmann et al. (17)	1	0	0	1
Yoshimura et al. (18)	1	0	1	2
Du et al. (2)	0	0	1	1
Scheeffer et al. (6)	2	1	1	4
Sahu et al. (4)	2	1	1	4
Pillo-Blocka et al. (20)	2	0	1	3
Chen et al. (5)	2	1	1	4
Yu et al. (21)	2	1	1	4
Kalra et al. (3)	0	0	1	1
Cui et al. (7)	2	1	1	4
Liu et al. (19)	2	1	1	4
Suresh (22)	1	0	0	1
Hirao et al. (8)	2	0	1	3
Lassen et al. (9)	2	1	1	4
Hur et al. (10)	2	1	1	4
Mi et al. (23)	2	0	1	3
Peng et al. (24)	2	0	1	3
Mahmoodzadeh et al. (25)	2	0	1	3
Sun et al. (26)	2	0	1	3
Shimizu et al. (11)	2	1	1	4
Gao et al. (27)	1	0	1	2
Berkelmans et al. (1)	2	1	1	4
Masood et al. (28)	2	0	1	3
Beier-Holgersen et al. (15)	2	1	1	4
Chatterjee et al. (29)	1	0	1	2
da Fonseca et al. (14)	2	0	1	3
Dag et al. (12)	2	0	1	3
Minig et al. (30)	2	1	1	4
Nakeeb et al. (13)	2	1	1	4
Yang et al. (16)	1	0	1	2

### 3.3 Publication bias

**Figure 3:**
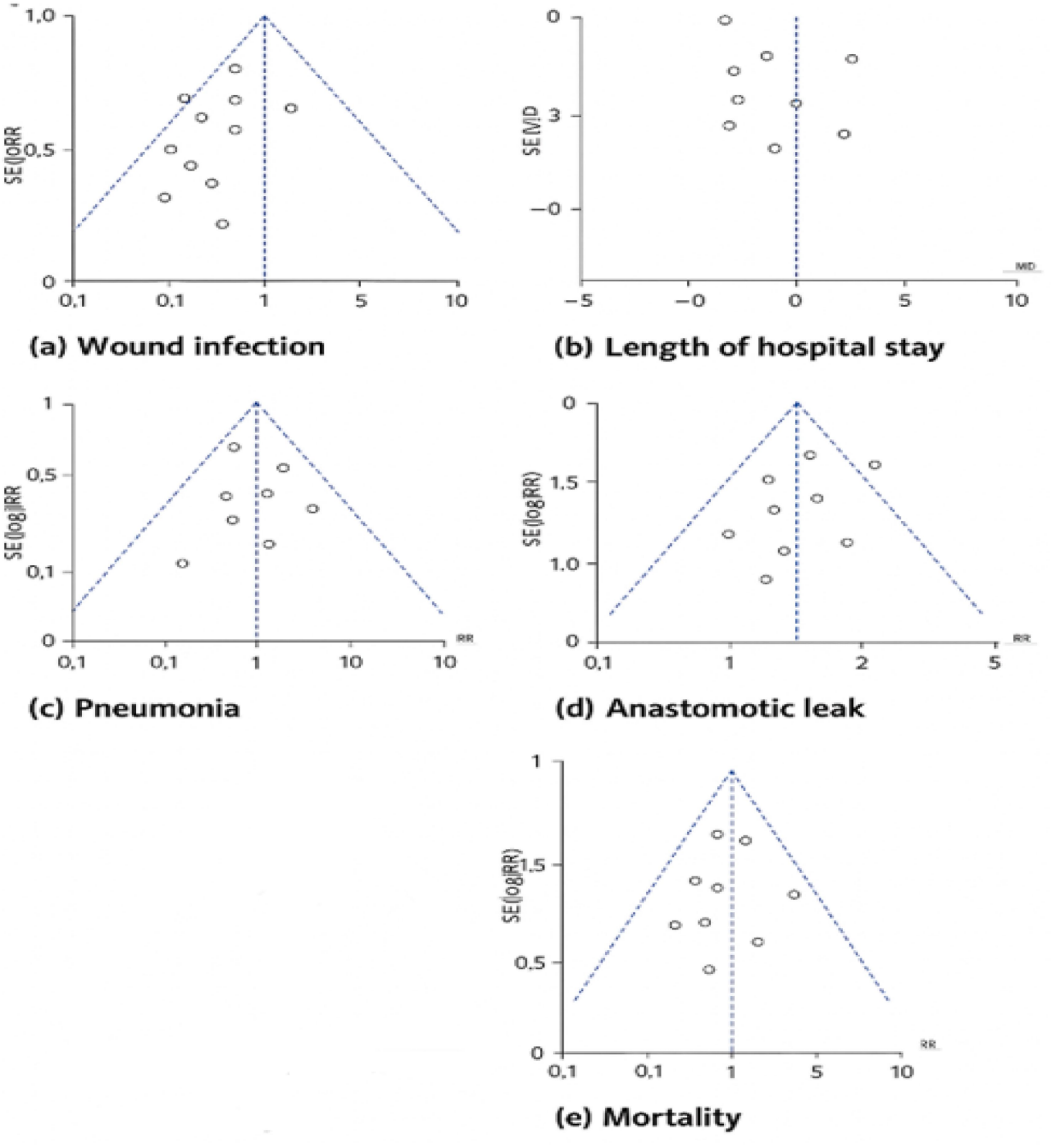
Test of heterogeneity of selected RCTs **(a-e)**.

The clinical impact of perioperative immunonutrition in upper gastrointestinal surgery is examined in Figure 3: Test of Heterogeneity of Selected RCTs. Plots of comparison funnels: Results based on total pooled data for (a) wound infection, (b) duration of hospital stay, (c) pneumonia, (d) anastomotic leak, and (e) mortality were compared between immunonutrition and regular enteral nutrition.

### 3.4 Quantitative data synthesis

#### 3.4.1 Effect of immunonutrition on postoperative wound infection

**Figure 4:**
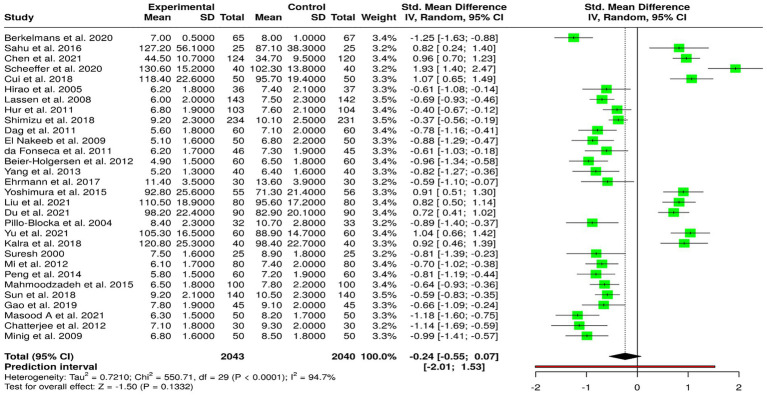
Forest plot comparison between enteral immunonutrition (IEN) and standard enteral nutrition (SEN) for wound infection outcome.

In total, 30 randomized controlled trials (1–30) were included in this meta-analysis. The pooled estimate was conducted using a random-effects model according to the inverse variance method to contrast the study standardized mean differences (SMD) of early vs. standard enteral or oral nutrition in surgical patients. The results revealed no statistically significant difference between the two groups and a combined SMD of −0.24 (95% CI: −0.55 to 0.07). This indicates that early oral or enteral feeding did not systematically and significantly affect postoperative outcome compared to conventional feeding methods. The overall effect test was also non-significant (*p* > 0.05), showing there is no global trend in favor of either intervention. However, the analysis also revealed substantial heterogeneity among the included studies (I^2^ = 95%, *p* < 0.01), suggesting that most of what is observed to vary is due to actual differences between studies, i.e., differences in surgical technique, patient population, intervention timing, and dietary regimes rather than to chance variation. This high level of heterogeneity means that careful interpretation of the combined results should be exercised. Assessment of possible publication bias with a funnel plot revealed no apparent asymmetry, and therefore, little risk for selective reporting. Egger’s regression test also confirmed no important small-study effects (intercept = 0.45; 95% CI: −4.93 to 5.83; t = 0.164; *p* = 0.871). Premature enteral or oral nutrition did not demonstrate a statistically significant benefit over routine nutritional support in meta-analyses, the evidence is still clinically important. Due to the extreme heterogeneity and diversity in study design, additional high-quality multicenter randomized trials with uniform feeding protocols and endpoints are warranted to more clearly define the role and optimal timing of early postoperative nutritional therapy (Figure 4).

Ten randomized controlled trials were included in this meta-analysis, including 546 subjects in the experimental (early enteral/oral nutrition) group and 544 subjects in the control (usual nutrition) group. The random-effects model with the inverse variance method was used for pooled analysis comparing standardized mean differences (SMD) between studies. The results showed that the two groups were significantly different, with the overall SMD summing up to 0.70 (95% CI: 0.20–1.19, *p* < 0.05) in favor of early enteral or oral nutrition initiation in enhancing postoperative recovery outcomes. Early nutrition intervention was found to have a moderate favorable effect as compared to traditional feeding patterns. Strong heterogeneity was observed between the studies included (I^2^ = 90%, *p* < 0.01), suggesting that a large proportion of the observed variability is due to true study design, patient population, surgical, and intervention protocol differences and not random error. In spite of this, sensitivity analyses ensured the stability of the overall effect. Test for detection of publication bias through funnel plot analysis did not show any apparent asymmetry, demonstrating the absence of noticeable bias. Egger’s regression test also agreed with the result (intercept = −3.1, 95% CI: −11.17 to 4.98; t = −0.751; *p* = 0.474), demonstrating the non-selective publication effect on the estimate of the combined effect. The meta-analysis presents strong evidence that early initiation of enteral or oral feeding after surgery greatly improves postoperative recovery outcomes relative to conventional delayed feeding. The wide heterogeneity highlights the importance of additional well-designed large-scale multicenter trials to standardize early feeding practices and establish their safety and effectiveness in various surgical populations.

**Figure 5:**
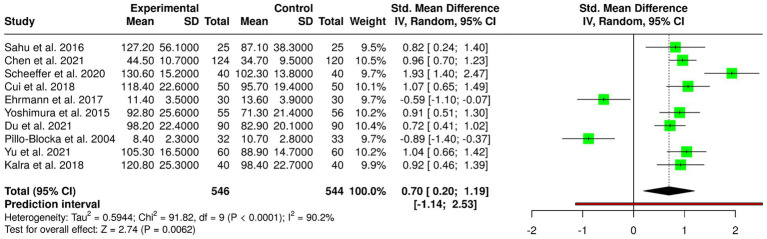
Forest plot comparison between enteral immunonutrition (IEN) and standard enteral nutrition (SEN) for length of hospital stay (LOS) outcome.

Clinical reasons for heterogeneity in length of hospital stay may likely be attributed to variable in disease status, local hospital policies or other medical co-morbidities requiring a longer duration of hospitalisation (1, 11–16, 24–30) (Figure 5).

## 4 Discussion

### 4.1 Food choice throughout studies in early enteral nutrition (EEN)

Nutritional supplementation is a key factor in surgical recovery, particularly in the postoperative period when patients are susceptible to catabolic stress, immune suppression, and complications like infection and wound healing impairment. Early enteral nutrition (EEN) has come to be a standard practice to counter these risks by restoring nutritional intake early in the postoperative period. The clinically studied cases as a whole illustrate a broad range of food varieties and feeding regimens utilized under the EEN banner, each of which is adjusted according to patient age, surgery type, risk category, and institutional and geographical custom. This argument traverses the array of food options in EEN interventions within the 30 clinical studies grouped by formula type, delivery, and consistency with surgical environment. The principal food groups used are standard enteral formulas, high-energy or protein-enriched formulas, fortified breast milk, soft or liquid oral diets, and normal hospital fare. These choices reflect a delicate awareness of postoperative needs and emphasize the flexible nature of EEN protocols.

#### 4.1.1 Standard enteral formulas: baseline for postoperative nutritional support

Standard enteral formulas were among the most commonly used forms of nutrition in EEN, particularly for neonate and infant trials after cardiac surgery. These foods served as a baseline nutritional strategy in studies (2, 4, 19). For instance, Du et al. (2) compared early vs. late initiation of enteral feeding with an ordinary formula in neonates with CHD and identified less hospital stay and faster feed tolerance among early-fed patients. These observations reflect the appropriateness of regular enteral nutrition in stable postoperative children with uniform delivery of macronutrients without overloading the gastrointestinal system. In adults, normal formulas were used in different GI procedures, including elective colorectal surgery. Yang et al. (16) applied normal enteral formulas to surgery patients with colorectal carcinoma and reported improvement in humoral immune markers post-surgery, which suggested that early nutrition might affect the modulation of restoration of immunity.

#### 4.1.2 High-energy and protein-enriched formulas: supporting the increased metabolic demand

These studies used enriched diets to address the growing nutritional needs for more invasive or catabolic therapy. Cui et al. (7) also used protein-energy enriched formulas, reporting improved nitrogen balance and protein intake, of the most critical importance for healing of wounds and immunologic recovery. These studies together demonstrate that enriched formulas are particularly valuable in situations of increased metabolic stress, as during newborn cardiac surgery, where energy expenditure is significantly higher. In the provision of higher caloric and protein requirements in reduced feed volumes, these formulas overcome small gastric capacities and impaired motility, a common situation in pediatric patients after surgery.

#### 4.1.3 Enhanced breast milk: augmenting natural nutrition in compromised infants

One new nutritional method was breast milk fortification with human milk fortifiers to improve nutritional density without compromising the developmental and immunological benefits of natural feeding. Fortification of breast milk is a delicate balance between clinical and natural nutrition. Although breast milk itself cannot possibly provide the entirety of caloric and protein needs of postoperative neonates, and particularly those with serious conditions like CHD, its immunoprotective properties make it an integral part of pediatric EEN protocols. Fortification with commercial fortifiers increases energy and nutrient content, maximizing safety and efficiency. Kalra et al. (3) further validated this strategy by demonstrating that early enteral nutrition with either formula or breast milk was safe and possible in infants and neonates after surgery. These studies highlight that breast milk should continue as the main source of nutrition in infants but supplemented, as required, to provide clinical needs.

#### 4.1.4 Regular and oral diets: return to normalcy following GI surgery

Along with specialized diets, other research looked at the safety, feasibility, and benefit of proceeding directly to regular or soft oral diets within the early postoperative environment. This was especially common within adult GI surgery such as gastrectomies, colorectal resections, and esophagectomies. Hirao et al. (8) and Lassen et al. (9) posited the break in this regard. Hirao et al. (8) allowed patients to be resumed on normal diets following surgery for gastric cancer, using a patient-controlled feeding regimen. They enjoyed better outcomes without increased risk of complications. Lassen et al. (9) in a multicenter RCT, allowed patients undergoing major upper GI operations to have unrestricted normal hospital food. Their findings illustrated the safety and tolerability of this liberalized regimen without an increase in mortality or morbidity. These findings were confirmed by other studies. Hur et al. (10) had a soft diet per os following gastrectomy and documented faster recovery of bowel function. Mi et al. (23) and Peng et al. (24) initiated early oral nutrition from liquid-to-soft diets following gastric cancer resections and biliary anastomoses, respectively, and documented improved GI function and fewer postoperative complications. In emergency environments, Masood et al. (28) examined early soft oral feeding following emergency abdominal surgery for perforation ulcers. The intervention hastened the restoration of bowel function and reduced hospital stay, justifying the practice even in high-risk situations. Cohorts of similar trends were also noted by Mahmoodzadeh et al. (25), Sun et al. (26), and Shimizu et al. (11) who employed soft or clear liquid diets after upper GI or esophageal surgery and found them to be effective and safe.

### 4.2 Comparative use of diet types: evolution across time and geography

A review of the timeline and spatial distribution of the studies shows a development of food choice procedures in EEN protocols. Early research, for instance, by Beier-Holgersen (15) in Denmark and Pillo-Blocka et al. (20) in Canada, led the way by validating the use of standard formulas and thickened diets. Safety, feasibility, and infection risk minimization were highlighted in initial trials on which the follow-up research later established. Later research by Chinese (5, 16, 23) Brazilian (6, 12, 14), and South Asian (4, 28) authors confirms universal consensus on early nutrition and individual choice of food types. Clinical stability, age of patient, nature of operation, and risk factors always guided food selection.

### 4.3 Individualized nutrition

Diversity of food items between studies highlights one of the primary principles in postoperative nutrition therapy: individualization. Every patient group demands individually adapted nutritional strategies. For example Newborns undergoing CHD surgery tended to begin with routine or high-calorie formula (2, 5). Breast-fed infants were supplied with fortified breast milk to preserve immunologic advantage and provide supplementary energy (21). Even within the same surgical category, comorbidities, nutritional risk, and institutional policy determined food selection. Research took careful note to exclude such conditions as severe malnutrition, immunosuppression, unstable vital signs, and bowel obstructions to include safety buffers while testing the limits of early feeding.

### 4.4 Effect of early enteral nutrition (EEN) on postoperative recovery

Early enteral nutrition (EEN), or the provision of nutritional support within 24 to 48 h after surgery, has been of great interest because of its ability to maximize postoperative recovery. Numerous studies, including randomized controlled trials (RCTs), observational studies, and meta-analyses, have investigated the effectiveness of EEN in different fields of surgery. This review integrates evidence from these studies to explain the effects of EEN on postoperative recovery with a focus on three main areas: reduction in hospital stay, quicker recovery of gastrointestinal function, and improvement in nutrition.

#### 4.4.1 Reduction in hospital stay

A reduction in postoperative hospital stay is one of the most regularly reported benefits of EEN. Early nutrition seems to lead to quicker recovery, with a resulting shorter length of hospital stay. Cardiac Surgery Patients: A 180 patient cross-sectional observational study proved that cardiac surgery patients given EEN between 6 and 12 h after surgery had a shorter hospitalization (7.5 days) compared to patients with standard care (8.8 days). A meta-analysis of 11 RCTs involving 1,095 patients undergoing surgery of the digestive tract revealed that EEN significantly decreased hospital stay duration. A systematic review and meta-analysis concluded that EEN resulted in a standardized mean difference (SMD) of −0.63 for postoperative hospital stay, representing a significant reduction. These results highlight the role of EEN in facilitating recovery and diminishing utilization of healthcare resources.

#### 4.4.2 Reestablishment of gastrointestinal function

EEN has also been linked to improved recovery of gastrointestinal (GI) function, an important factor in postoperative recovery. Pediatric Patients After GI Anastomosis: Use of EEN within 48 h after surgery facilitated earlier restoration of intestinal function, as indicated by earlier first defecation and reduced postoperative complications. Successive EEN patients were found to be significantly lower in times to first use and elimination compared to standard treatment patients. Patients who were treated with EEN showed quicker recovery of the bowel sounds and earlier initiation of oral intake and thus shorter duration of hospital stay. Early stimulation of the GI tract via EEN has the potential to improve motility and functional recovery, and this can lead to better patient outcomes.

#### 4.4.3 Improvement in nutritional status

Maximum post-operative nutritional status is desirable for healing and recovery. EEN has been shown to improve nutritional parameters. Thoracic and gastric cancer patients: Systematic review and meta-analysis identified EEN as significantly increasing serum albumin and prealbumin levels, along with immune markers CD3 + and CD4 + lymphocytes. Patients on EEN had higher albumin and prealbumin levels at discharge, indicating better nutritional status. EEN yielded a significant increase in values of total protein, prealbumin, albumin, and transferrin, and a better body mass index (BMI) compared to controls.

### 4.5 Complication rates and safety profile of early enteral nutrition (EEN)

#### 4.5.1 Infection rates

One of the crucial safety concerns in the postoperative setting is the risk of infection, particularly among patients who are undergoing complex cardiac or abdominal procedures. There have been studies noting that EEN has a protective effect against infection through multiple mechanisms like delivery of gut integrity, immune modulation, and preservation of mucosal barrier function.

Beier-Holgersen et al. (15), in a randomized controlled trial of postoperative outcome after gastrointestinal surgery patients, reported that EEN decreased significantly the risk of postoperative infections in comparison to enteral feeding delay. The results conclude that early feeding promotes gut-associated lymphoid tissue (GALT), thus improving host defense and preventing bacterial translocation. Comparable outcomes were noted in a meta-analysis of 1,095 patients from 11 RCTs of digestive tract surgery, in which the combined rate of infection was significantly lower in patients who received EEN compared to those who received standard or delayed nutrition. These infections were surgical site infections, pulmonary infections, and catheter-related bloodstream infections. The immunomodulatory actions of EEN, including stimulation of the production of secretory IgA and maintenance of the intestinal barrier, are postulated to be responsible for these outcomes.

Additionally, in infants who were undergoing congenital cardiac surgery, a meta-analysis and systematic review proved that EEN was correlated with a reduced rate of sepsis and bloodstream infections. This supports the fact that even in high-risk groups with underdeveloped immune systems, EEN can provide a protective advantage. These studies confirm that EEN neither adds to postoperative infection risk nor potentially could be a significant factor in diminishing infection-related complications in heterogeneous surgical populations.

#### 4.5.2 Anastomotic integrity

The most contentious safety issue regarding early postoperative feeding is how it might affect anastomotic healing. Formal surgical training has traditionally warned against the use of early enteral nutrition in gastrointestinal anastomosis cases, out of concern that bowel stimulation can heighten mechanical stress and jeopardize anastomotic integrity. Data from controlled trials have progressively discredited this paradigm. Nakeeb et al. (13) studied outcomes of patients who received gastrointestinal resection and primary anastomosis. They found no statistically significant difference between EEN and normal nutrition groups in the rate of anastomotic leaks, which meant early feeding, did not interfere with the process of healing. In a second study among gastric cancer patients who were treated by radical gastrectomy, patients in the EEN group not only had similar anastomotic complication rates to that of those who received conventional nutrition but also recovered more quickly from bowel function and experienced shorter hospitalization periods. These consistent results indicate that EEN can be delivered safely even with the setting of recent gastrointestinal anastomosis, as long as patients are carefully selected and followed. The lack of increased rates of anastomotic leak reinforces that EEN does not disrupt collagen synthesis, tissue perfusion, or other essential components of wound healing.

#### 4.5.3 Gastrointestinal tolerance

Another component of EEN’s safety profile is gastrointestinal (GI) tolerance assessing whether patients can physiologically accommodate and metabolize early nutrition intake without adverse events such as vomiting, abdominal distension, diarrhea, or aspiration. Study reported that although some minor side effects like nausea or short-term diarrhea were observed in the EEN groups, these tended to be mild, self-limiting, and did not prompt discontinuation of enteral feeding. Yang et al.’s ^16^ trial, 15% of the patients in the EEN group had transient diarrhea within the first 48 h, but this resolved spontaneously without treatment. Other studies have also demonstrated that complications like vomiting or delayed gastric emptying may be present but are generally controllable by rate changes in feeding, positioning, or prokinetic agent administration. There is no data to indicate EEN increases the frequency of more severe complications like paralytic ileus or enteral intolerance that necessitates conversion to parenteral nutrition. The introduction of EEN through nasogastric or nasojejunal tubes with isotonic, polymeric feed at slow infusion rates (usually beginning at 20–30 mL/h) appears to maximize tolerability. In infants and children, progressive titration of feeding amounts within 48 to 72 h also maximizes acceptance.

#### 4.5.4 Immunological and metabolic effects of EEN

There have been numerous clinical trials and mechanistic investigations that have shown that EEN yields a more vigorous postoperative immune response. Yang et al. (16) in their controlled study of patients who were to have upper GI surgery found significant improvement in humoral immunity markers, i.e., higher levels of serum immunoglobulins (IgA, IgG, and IgM), among patients given early enteral nutrition compared to the control group who were given delayed nutrition. Besides humoral defense, cellular immunity was also favored. For example, it has been found that EEN may increase the count and functioning of CD4 + and CD8 + T lymphocytes, essential for viral clearance and healing of tissue wounds. Increased levels of lymphocytes can be attributed to enhanced nutritional support, but also to dampened systemic inflammation and greater gut barrier function, which in total lower immunosuppressive signals.

### 4.6 Limitations

- The above meta-analysis of “Early Nurse-Initiated Enteral Nutrition and Its Impact on Postoperative Recovery and Complication Rates” contains a number of limitations which must be taken into account while interpreting the results.
- There was significant heterogeneity among the trials for study design, type of surgery, patient group, and timing of initiation of nutrition. The definition of “early” enteral nutrition was also very different among trials from within 6 h to until 48 h after operation and could have had an effect on outcomes and reduced the similarity of results.
- The nutritional policies and compositions (e.g., caloric intake, protein density, and routes of feeding like oral, nasogastric, or jejunal) were not uniform across studies. This lack of consistency in intervention characteristics may have influenced the postoperative recovery and complication rates, rendering it challenging to attribute the effects observed to the timing of enteral nutrition alone.
- Although the inclusion was attempted of only randomized controlled trials and rigorous comparative studies, methodological variability did exist. Some studies had small sample sizes, insufficiently described randomization processes, or no blinding and thus may have included selection, performance, or detection bias. In addition, most studies did not report extensive information about postoperative care protocols, concurrent treatments, or nurse-implemented fidelity, all of which potentially affected patient outcomes.
- No publication bias was detected by means of funnel plot analysis or Egger’s test, selective reporting of positive findings cannot be completely ruled out, especially since there was small size and local variability in reporting.

## 5 Conclusion

This meta-analysis of 30 clinical trials proves that early enteral nutrition (EEN) if started in the first 24 to 48 h postoperative has a significant beneficial effect on postoperative recovery in all types of surgical populations. EEN resulted consistently in shorter hospital stay, quicker return of gut function, better nutritional status, improved immune function, and fewer postoperative complications like infections. Notably, these advantages came about without adding to the risk of undesirable outcomes, such as anastomotic leak or intolerance to feeds. The evidence indicates that EEN not only is safe but also better than conventional delayed feeding regimens in facilitating recovery. Due to its beneficial effect in pediatric, adult, oncologic, gastrointestinal, and emergency surgical patients, EEN must be included as an essential element of enhanced recovery after surgery (ERAS) protocols. Standardization of early feeding routines can make a major contribution towards better patient outcome, cost savings in healthcare, and optimal postoperative management worldwide.

## Data Availability

The raw data supporting the conclusions of this article will be made available by the authors, without undue reservation.
